# The Multimodal Concept of Hemodynamic Stabilization

**DOI:** 10.3389/fpubh.2014.00034

**Published:** 2014-04-30

**Authors:** Krisztián Tánczos, Márton Németh, Zsolt Molnár

**Affiliations:** ^1^Department of Anaesthesiology and Intensive Therapy, University of Szeged, Szeged, Hungary

**Keywords:** hemodynamic optimization, cardiac output, stroke volume, central venous oxygen saturation, venous to arterial carbon dioxide gap

## Abstract

Hemodynamic instability often leads to hypoperfusion, which has a significant impact on outcome in both medical and surgical patients. Measures to detect and treat tissue hypoperfusion early by correcting the imbalance between oxygen delivery and consumption is of particular importance. There are several studies targeting different hemodynamic endpoints in order to investigate the effects of goal-directed therapy on outcome. A so-called multimodal concept putting several variables in context follows simple logic and may provide a broader picture. Furthermore, rather than treating population based “normal” values of certain indices, this concept can be translated into the individualized patient care to reach adequate oxygen supply and tissue oxygenation in order to avoid under, or over resuscitation, which are equally harmful. The purpose of this review is to give an overview of current data providing the basis of this a multimodal, individualized approach of hemodynamic monitoring and treatment.

## Introduction

Development of multiorgan disorders is often the result of hypoperfusion, which severely affects outcome of medical and surgical patients alike and substantially increases the utilization of resources and costs (1). Therefore, the use of early and efficient therapeutic strategies able to detect tissue hypoperfusion and to treat the imbalance between oxygen consumption and delivery is of particular importance (2). Traditional endpoints such as heart rate, blood pressure, mental status, and urine output can be useful in the initial identification of inadequate perfusion, but are limited in their ability to identify ongoing, compensated shock (3). Therefore, more detailed assessment of global macrohemodynamic indices such as cardiac output (CO) and derived variables and measures of oxygen delivery and uptake, may be necessary to guide treatment (4, 5). Furthermore, after the optimization of these parameters, indicators of tissue perfusion should also be assessed to verify the effectiveness of therapy (6).

## Physiological Issues

**Figure 1:**
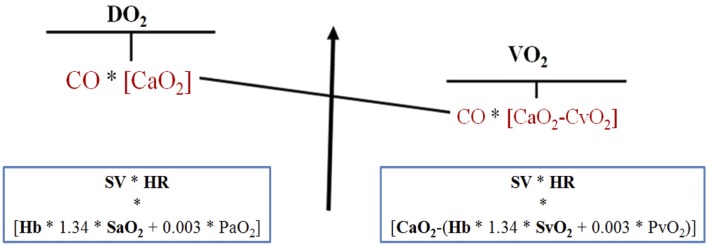
**Oxygen delivery and consumption**. DO_2_, oxygen delivery; SV, stroke volume; HR, heart rate; Hb, hemoglobin; SaO_2_, hemoglobin arterial oxygen saturation; PaO_2_, arterial oxygen partial pressure; CO, cardiac output; CaO_2_, arterial oxygen content, VO_2_; oxygen consumption, SvO_2_; hemoglobin mixed venous oxygen saturation; PvO_2_, venous oxygen partial pressure; CvO_2_, venous oxygen content.

The primary goal of the cardiorespiratory system is to deliver adequate oxygen to the tissues to meet their metabolic requirements. The adequacy of tissue oxygenation is determined by the balance between the rate of oxygen transport to the tissues (oxygen delivery, DO_2_) and the rate at which the oxygen is used by the tissues (oxygen consumption, VO_2_) (7). The standard formulas to determine oxygen delivery and oxygen consumption is shown in Figure 1.

In the critically ill and in the perioperative period, there is often an imbalance between delivery and consumption. Oxygen delivery can be inadequate if arterial oxygen content (CaO_2_) and/or CO is reduced (8, 9). The circulation can compensate to some extent, and VO_2_ is usually independent in a wide range of DO_2_. However, beyond a critical point any further drop in DO_2_ will inevitably result in a decrease in VO_2_. In other words, after exhausting compensatory resources VO_2_ becomes dependent on DO_2_ and aerobic metabolism will have to be switched to anaerobic metabolism, leading to metabolic acidosis and oxygen debt (10).

The principle task of acute care is to avoid or correct oxygen debt by optimization of the oxygen supply and consumption. Furthermore, it is just as important to recognize that DO_2_ and tissue perfusion has normalized, therefore any further measures to increase DO_2_ may do harm by unnecessary over resuscitation.

There is also mounting evidence that conventional parameters such as blood pressure, central venous pressure, heart rate are poor indicators of cardiac index or oxygen delivery (11, 12), and there is also increasing evidence that, for example, in high-risk surgery perioperative care algorithms based on advanced hemodynamic monitoring are beneficial (13, 14).

## Goal-Directed Concept in Hemodynamic Monitoring

The multimodal concept in hemodynamic monitoring can be translated into the individualized use of target endpoints for hemodynamic stabilization instead of treating “normal” values, and can help to reach adequate oxygen supply and tissue oxygenation in order to avoid under or over resuscitation, which are equally harmful. It is important to note, that so-called “normal” values may be true for a population, but may be false for an individual patient.

### Cardiac output and DO_2_ as resuscitation endpoints

Several clinical investigations were performed on CO and derived variables based goals directed hemodynamic support in high-risk surgery. In two recent meta-analyses, it was found that cardiac index and DO_2_ guided treatment resulted in reduced mortality as compared to high-risk surgical patients receiving standard therapy (13, 14).

### Stroke volume variation and pulse pressure variation as resuscitation endpoints

Recently, less invasive devices assessing CO by pulse contour analysis based on the radial artery pressure signal have been introduced. Although these devices show lower precision compared to the gold standards of thermodilution, there is some evidence that these methods can adequately show changes and trends in the hemodynamic status (15). As pulse pressure variation and stroke volume variation are well established indicators of fluid responsiveness, these devices seem to be simple and useful alternatives to invasive hemodynamic monitoring (16). Furthermore, in recent studies fluid therapy guided by SVV and PPV proved to be more accurate than static preload indicators-based approaches and has also been shown to improve patient outcome, by reducing postoperative complication rate significantly (17, 18). However, pulse pressure variation and stroke volume variation are limited to patients who receive controlled mechanical ventilation with normal sinus rhythm (19, 20).

### Venous to arterial CO_2_ gap as therapeutic endpoint

Another easily obtainable blood flow related blood gas parameter is the central venous to arterial carbon dioxide gap (dCO_2_). Several authors have reported increased dCO_2_ in different low flow states (21–23). In oxygen debt caused anaerobic metabolism, hydrogen ions are generated in two ways: (1) hydrolysis of adenosine triphosphate to adenosine diphosphate and (2) increased production of lactic acid (24). Hydrogen ions are buffered by bicarbonate presented in the cells, and this process will generate CO_2_ production (25). While arterial PaCO_2_ is variable and dependent on pulmonary gas exchange, central venous PvCO_2_ is dependent on the capability of the flow (i.e., CO) to wash out carbon dioxide from the tissues. The Fick principle adapted to carbon dioxide demonstrates the inverse relationship between the CO and dCO_2_ (26). This postulate that increased dCO_2_ reflects decreased flow was confirmed in several critically ill conditions such as severe sepsis, heart failure, and severe hypovolemia (27, 28). Furthermore, adding the dCO_2_ to ScvO_2_ for identifying VO_2_/DO_2_ >30%, there was an improvement in specificity, positive predictive, and negative predictive values (29).

In cases like severe sepsis, when oxygen uptake is insufficient due to microcirculatory and/or mitochondrial defects, ScvO_2_ may be elevated (i.e., false negative). Previous studies have suggested that under such circumstances the increased value of dCO_2_ (>5 mmHg), may help the clinician in detecting inadequate DO_2_ to tissues, hence the complementary use of ScvO_2_ and dCO_2_ is recommended (30–32).

### Measures of oxygen delivery and extraction

Perhaps the most commonly used methods to assess global VO_2_/DO_2_ are mixed venous oxygen saturation (SvO_2_) and its surrogate ScvO_2_. Central venous oxygen saturation is an easily obtained parameter via a central venous catheter already *in situ* in most critically ill patients and it is often used as a marker of the balance between oxygen delivery and consumption. Because of the different positions of the pulmonary artery and central venous catheters (entire body in the case of SvO_2_ versus brain and the upper part of the body in the case of ScvO_2_) there has been a considerable debate on the interpretation of ScvO_2_ values as compared to SvO_2_. Most of the studies that have analyzed the relationship between ScvO_2_ and SvO_2_ have shown that ScvO_2_ is on an average 5% higher than SvO_2_ and is considered as a reasonable surrogate marker in the clinical setting (33–35). However, recent clinical trials, mainly on septic patients, were unable to show satisfactory agreement between ScvO_2_ and SvO_2_. This could in part be explained by modifications of blood flow distribution and oxygen extraction by brain and splanchnic tissues (36). It seems that ScvO_2_ and SvO_2_ are not numerically equivalent but the changes usually occur in a parallel manner (37).

The main factors, which influence ScvO_2_, are hemoglobin, arterial oxygen saturation of hemoglobin, CO, and oxygen consumption. Theoretically if three of these factors are kept constant, the value of ScvO_2_ reflects the changes of the latter. There are multiple physiologic, pathologic, and therapeutic factors, which influence venous oxygen saturation, such as anemia, hypovolemia, contractility, bleeding, sedation, fever, pain, etc. (38).

One of the important features of venous saturation is that it can be pathologic both when it is high and when it is low. In a recent large cohort of septic patients in the emergency department, it was found that mortality was 40% in patients admitted with an ScvO_2_ <70% but in patients with an initial ScvO_2_ of >90%, it was almost as high 34%. The latter was probably due to impaired oxygen utilization (39). High ScvO_2_ values may thus represent an inability of the cells to extract oxygen or microcirculatory shunting in sepsis (40). Therefore, additional measures are necessary to help evaluating high ScvO_2_ values, such as for example lactate, central venous to arterial dCO_2_, and by applying advanced invasive hemodynamic monitoring.

Lactate, the end product on anaerobic metabolism, has been thoroughly investigated over the last decades in critical care. It has good prognostic value in several clinical scenarios such as trauma, sepsis, and high-risk surgical patients (41). Not just the absolute value, but its change over time (kinetics: determined by production and clearance) seems an even better marker of adequate resuscitation and outcome (42). A lactate decrease by 20% or more per 2 h in the initial resuscitation of critically ill patients resulted shorter length of stay in the intensive care unit and a lower mortality rate when adjusted to predefined risk factors (43). However, if lactate kinetics is assessed every 2–6 h, which can be regarded as far too long considering that acute resuscitation should be corrected as soon as possible, it seems that lactate kinetics rather than absolute values should be followed as resuscitation endpoints. In cases, when lactate production or elimination is impaired, the evaluation of lactate clearance is difficult to interpret. These pathological circumstances can be liver failure (44) or seizures (45).

### PPV, dCO_2_, and stroke volume guided fluid resuscitation

In a recent animal experiment, we tested the effect of stroke volume guided hemorrhage and fluid resuscitation (46). After baseline measurements (*T*_bsl_), animals were bled until stroke volume index dropped by 50%, then measurements were repeated (*T*_0_). Thereafter animals were resuscitated with lactated Ringer’s solution until baseline SVI values were reached, then final measurements were recorded (*T*_end_). After bleeding, the SVI decreased by the planned 50% at *T*_0_ and returned to its initial value by *T*_end_ (Table 1). The CI also decreased after bleeding and reached a higher value by *T*_end_ as compared to *T*_bsl_. Pulse contour analysis driven SVV and PPV increased from *T*_bsl_ to *T*_0_ and normalized by *T*_end_. ScvO_2_ decreased from *T*_bsl_ to *T*_0_ and although increased by *T*_end_, it remained lower, with a mean difference of 5% as compared to *T*_bsl_.

**Table 1:** **Hemodynamic. and blood gas changes during stroke volume based fluid resuscitation**.

	*T*_bsl_	*T*_0_	*T*_end_
Stroke volume index (mL/m^2^)	26.8 ± 4.7	13.4 ± 2.3*	26.6 ± 4.1^#^
Cardiac index (L/min/m^2^)	2.6 ± 0.4	1.8 ± 0.3*	2.9 ± 0.5*^,#^
Stroke volume variation (%)	13.6 ± 4.3	22.6 ± 5.6*	12.2 ± 4.3^#^
Pulse pressure variation (%)	13.0 ± 4.5	24.5 ± 7.6*	13 ± 4.2^#^
Venous to arterial carbon dioxide gap (mmHg)	5.3 ± 2	9.6 ± 2.3*	5.1 ± 2.6^#^
Central venous oxygen saturation (%)	78 ± 7	61 ± 5*	73 ± 9*^,#^
Hemoglobin (g/dL)	12.05 ± 1.37	11.22 ± 1.39*	8.45 ± 1.1*^,#^

Data are expressed as mean ± SD; *p < 0.05 significantly different from *T*_bsl_; ^#^*p* < 0.05 significantly different from *T*_0_.

**T*_0_, baseline measurements; *T*_1_, measurements following the hemorrhage; *T*_end_, measurements after the resuscitation. Data are presented as mean ± SD, statistically significant difference was considered *p* < 0.05*.

*Significantly different from *T*_0_.

*^#^Significantly different from *T*_end_*.

In these experiments, ScvO_2_ and dCO_2_ correlated well with changes in stroke volume. If the hemodynamic instability is corrected, stroke volume, PPV, SVV, and dCO_2_ are in the physiological range, the low ScvO_2_ can indicate a low hemoglobin level due to low oxygen delivery. These data also confirm that more parameters should be taken into account during resuscitation.

## Conclusion

Early and adequate hemodynamic stabilization of the critically ill has a significant effect on outcome. Rather than following certain numbers in protocols or algorithms, a multimodal approach, of assessing hemodynamic variables together with the balance between oxygen delivery and consumption, may help to get a detailed picture about the hemodynamic status of our patients and also gives a chance for individualized treatment. The latter means that the evidence, which proved beneficial for a population in clinical studies gives the frame what we fine tune for the patient’s individual needs reflected by changes in this complex picture of physiology. Despite that this multimodal approach follows simple logic, it has currently not been completely proven, which renders the need for further clinical trials.

## Conflict of Interest Statement

The authors declare that the research was conducted in the absence of any commercial or financial relationships that could be construed as a potential conflict of interest.
